# Prion Diseases—When Proteins Turn Lethal: Creutzfeldt–Jakob Disease (CJD) and the Quest for Classification, Diagnosis, Therapeutic Approaches, and Emerging Research

**DOI:** 10.3390/molecules31081265

**Published:** 2026-04-11

**Authors:** Tamil Selvan Ramesh, Dorota Bartusik-Aebisher, Klaudia Dynarowicz, David Aebisher

**Affiliations:** 1 English Division Science Club, Faculty of Medicine, University of Rzeszów, 35-310 Rzeszów, Poland; tr131820@stud.ur.edu.pl; 2 Department of Biochemistry and General Chemistry, Faculty of Medicine, University of Rzeszów, 35-310 Rzeszów, Poland; dbartusikaebisher@ur.edu.pl (D.B.-A.); kdynarowicz@ur.edu.pl (K.D.); 3 Department of Photomedicine and Physical Chemistry, Faculty of Medicine, University of Rzeszów, 35-959 Rzeszów, Poland

**Keywords:** Creutzfeldt–Jakob disease, prion disorders, sCJD, vCJD, fCJD, iCJD, EEG in CJD, MRI in CJD, palliative care, CJD prognosis

## Abstract

Creutzfeldt–Jakob disease (CJD) is a rare and still fatal neurodegenerative disorder caused by prion protein misfolding in the central nervous system. Accumulation of the pathogenic isoform leads to neuronal damage, spongiform degeneration, and rapidly progressive dementia. The disease is divided into sporadic, familial, iatrogenic, and variant forms, with sporadic cases accounting for the majority of cases. Diagnosis remains challenging and relies on a combination of clinical assessment, neuroimaging, and laboratory biomarkers. Key diagnostic methods include electroencephalography, Magnetic Resonance Imaging, and cerebrospinal fluid analysis for proteins as well as advanced amplification tests that improve diagnostic accuracy. Despite these advances, early detection remains challenging and misdiagnosis can occur. Currently, there is no effective disease-modifying therapy, and treatment is primarily supportive, focusing on symptom control and palliative care. Ongoing research aims to better understand the molecular mechanisms underlying prion propagation and develop targeted therapeutic strategies. This review summarizes current diagnostic methods and therapeutic approaches, focusing on molecular applications and their potential clinical implications.

## 1. Introduction

Creutzfeldt–Jakob disease (CJD) is a rare, rapidly progressive, and still fatal neurodegenerative disease [1,2,3,4,5,6,7,8] belonging to the group of transmissible spongiform encephalopathies (TSEs) [9,10]. Unlike most neurodegenerative diseases, CJD is caused not by conventional pathogens such as viruses or bacteria, but by prions, misfolded isoforms of the host-encoded prion protein [11,12]. They have the unique ability to propagate by inducing conformational changes in their normal counterparts. This characteristic mechanism of protein misfolding and aggregation underlies a cascade of neurodegenerative processes, ultimately leading to widespread neuronal loss, spongiform degeneration, and severe cognitive decline [13]. Despite its low incidence, estimated at approximately 1–2 cases per million people per year, CJD poses a significant clinical challenge due to its rapid progression, diagnostic complexity, and lack of effective disease-modifying therapies [11]. The disease occurs in several forms—sporadic, familial, iatrogenic, and variant—that differ in etiology, epidemiology, and clinical presentation but share a common molecular basis derived from prion biology [13,14]. Among these, sporadic CJD accounts for the majority of cases, while variant CJD has attracted particular attention due to its association with bovine spongiform encephalopathy and its implications for zoonotic transmission. Advances in neuroimaging, cerebrospinal fluid biomarkers, and amplification tests such as real-time tremor-induced conversion (RT-QuIC) have improved the diagnosis of CJD [15,16]; however, early and accurate detection remains challenging. Clinical manifestations often overlap with other rapidly progressive dementias, contributing to potential misdiagnosis and delayed intervention [17,18]. Furthermore, current treatment strategies remain largely supportive, underscoring the urgent need for improved diagnostic tools and the development of targeted therapeutic approaches.

The aim of this review is to provide a comprehensive overview of CJD, with particular emphasis on its classification, molecular mechanisms, clinical presentation, diagnostic strategies and current therapeutic approaches, as well as new directions in prion research that may shape future clinical practice.

## 2. Materials and Methods

A thorough literature search was conducted using medical databases including PubMed, Scopus, Science Direct, Research gate and Web of Science. Data was also retrieved from governmental health websites, namely the Mayo Clinic, and the CJD Foundation. Inclusion criteria were peer-reviewed articles published in English within the last 25 years. Both clinical and experimental studies and reviews were taken into consideration. Studies focusing on diagnosis, subtype differentiation, and treatment outcomes and recent research were given priority.

Keywords such as “Creutzfeldt–Jakob Disease,” “prion disorders,” “sCJD,” “vCJD,” “fCJD”, “iCJD”, “EEG in CJD,” “MRI in CJD,” “palliative care”, and “CJD prognosis” were used. All figures and tables were created by the authors themselves. Tools and software such as Goodnotes (version 5), Procreate (version 5.4), and Microsoft Word (version 2021) were used.

## 3. Creutzfeldt–Jakob Disease Characteristic

**Figure 1:**
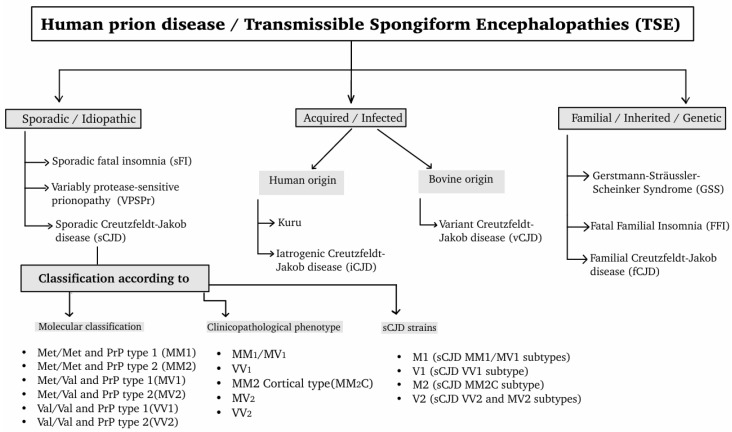
A flow diagram illustrating the Classification of Human prion diseases /TSEs (transmissible spongiform encephalopathy).

The disease was first described by Hans Gerhard Creutzfeldt in 1920 followed by Alfons Maria Jakob in 1921 and 1923 and later was called by the term “Creutzfeltd-Jakob Disease” by Clearance J. Gibbs [15,19,20]. Currently, Palliative care rather than curative is the only way of management of the disease. Sporadic CJD (sCJD) is the most common type of prion disease in humans [19]. The sCJD occurs when the normal prion protein (PrP^C^) encoded by chromosome 20 spontaneously misfolds into an abnormal isoform PrP^Sc^ [3,6,8]. Though there is no clear function of this protein, it may play a role in the metabolism of copper and antioxidant systems. The post-translational conformational change leads to the misfolding of the protein PrP^C^, resulting in the formation of a misfolded insoluble protein (PrP^Sc^), which then deposits in tissues as amyloid and is strongly associated with prion diseases [16]. The peak incidence is in the Seventh decade of life (70 s) but can vary according to the type of CJD. Age, genetic predisposition, medical and dietary exposure are a few known risk factors associated with the disease. Early diagnosis can be challenging and use of tools such as electroencephalography (EEG), Magnetic Resonance Imaging (MRI), cerebrospinal fluid (CSF) analysis, real-time quaking-induced conversion (RT-QuIC), and genetic testing can facilitate early diagnosis [15]. Human prion diseases, classified as transmissible spongiform encephalopathies (TSEs), constitute a heterogeneous group of neurodegenerative diseases that differ in etiology, clinical presentation, and molecular mechanisms. These diseases can be broadly divided into sporadic, acquired, and familial forms, each associated with distinct disease initiation pathways. Prion diseases—and sCJD in particular—can be further subdivided based on molecular features and clinicopathological phenotypes. This classification reflects the complexity of prion strain diversity and its impact on disease progression and clinical variability. The main categories and subtypes of human prion diseases are summarized in Figure 1.

The Human prion diseases are classified as sporadic, acquired and familial. Sporadic/idiopathic human prion diseases consist of sFI, Variably Protease-Sensitive Prionopathy (VPSPr), and sCJD. The sCJD can further classified according to molecular, clinicopathological phenotype and sCJD strains. MM1, MM2, VV1, VV2, MV1 and MV2 fall under molecular classification. MM1/MV1, VV1, MM2C, MV2, VV2 are classified under clinicopathological phenotype. The sCJD strains consist of M1, V1, M2, V2. Acquired/Infected human prion diseases can be either human (kuru, iCJD) or bovine origin (vCJD). Familial/inherited/genetic human prion diseases consist of Gerstmann–Sträussler–Scheinker Syndrome (GSS), Fatal familial insomnia (FFI), fCJD.

Sporadic Fatal Insomnia (sFI), Variably Protease-Sensitive Prionopathy (VPSPr), sporadic Creutzfeldt–Jakob disease (sCJD), MM1 (Met/Met and PrP type 1), MM2 (Met/Met and PrP type 2), MV1 (Met/Val and PrP type 1), MV2 (Met/Val and PrP type 2), VV1 (Val/Val and PrP type 1), VV2 (Val/Val and PrP type 2), MM2-cortical-type (MM2C), M1 (sCJD MM1/MV1 subtypes), M2 (sCJD MM2C subtype), V1 (sCJD VV1 subtype), V2 (sCJD VV2 and MV2 subtypes), GSS, Fatal familial insomnia (FFI), Familial CJD (fCJD).

Table 1 showing Prion diseases and their respective Prion protein gene (PRNP) mutations referred from ECDC [17]. It summarizes prion diseases associated with mutations in the PRNP gene, which encodes the prion protein (PrP), together with their corresponding neuropathological phenotypes. Individual disease entities differ in their clinical and pathological presentation, largely depending on the type and location of the mutation, including point substitutions, insertions, or deletions within the octapeptide repeat region.

This table summarizes known mutations in the prion protein gene (PRNP) and their associated neuropathological phenotypes. Listed are inherited and genetic prion diseases, including Gerstmann–Sträussler–Scheinker syndrome (GSS), Creutzfeldt–Jakob disease (CJD), and Fatal familial insomnia (FFI). The table also includes variants associated with vascular prion protein amyloid deposition and mutations of uncertain or not yet confirmed pathogenic significance linked to neuropsychiatric disorders. Mutations in the prion protein gene (PRNP) produce distinct clinicopathological phenotypes depending on how they influence protein misfolding and aggregation. In Gerstmann–Sträussler–Scheinker syndrome (GSS), mutations such as P102L, P105L, A117V, G131V, F198S, D202N Q212P, Q217R, M232T and 192 bpi promote the plaque deposition [plaque dominant phenotype]. In contrast, mutations associated with Creutzfeldt–Jakob disease (CJD), including D178N129V, V180I, V180I + M232R, T183A, T188A, E196K, E200K, V203I, R208H, V210I, E211Q, M232R, 96 bpi, 120 bpi, 144 bpi, 168 bpi and 48 bp del, typically destabilize the prion protein in a way that favors formation of diffuse, non-amyloid PrPˢᶜ aggregates, resulting in synaptic deposition and prominent spongiform degeneration rather than plaque formation [Spongiform phenotype]. The D178N mutation combined with valine at codon 129 (129V) specifically produces a CJD phenotype by stabilizing a CJD type strain. The same D178N mutation, when coupled with methionine at codon 129 (129M), instead causes Fatal familial insomnia (FFI), characterized by selective thalamic degeneration and minimal spongiosis [Thalamic phenotype], illustrating how codon 129 polymorphism determines strain behavior and brain targeting. Rare variants like Y145s lead to vascular amyloid deposition [Amyloid angiopathy], while others (H187R or 216 bpi) produce mixed/atypical phenotypes. Finally, several reported variants (I138M, G142S, Q160S, T188K, M232R, 24 bpi, 48 bpi, 48 bpi + nucleotide substitution in other octapeptides) are not definitively classified as prion diseases [no consistent prion pathology] because they lack consistent transmissibility, typical PrPˢᶜ accumulation, and characteristic neuropathological features.

## 4. Epidemiology

Four types of CJD have been described:

### 4.1. Sporadic CJD (sCJD)

sCJD (Table 2) is the most common form of CJD, making up about 85–90% of all cases [11,21,22,23]. It affects people aged 45 to 75 years. Symptoms begin to appear between the ages of 60 and 65. The exact cause of this type is mostly unknown. However, it can be caused by the spontaneous misfolding of the prion protein (PrP) in the brain. The progression of the disease is quick, and those affected generally pass away within 6 to 12 months after symptoms begin with a mean survival rate of four months [12,24]. The early signs mainly include memory loss/amnesia, decline in thinking ability/dementia, and loss of coordination and balance/ataxia. These symptoms quickly progress to myoclonus, vision problems, and overall weakness. Although sCJD is the most prevalent form of the disease, it remains quite uncommon, impacting only around one or two individuals per million annually [3,25].

The PrP^C^ is a membrane glycoprotein (glycosylphosphatidylinositol anchored) which is expressed on neural and non-neural tissues of the CNS (central nervous system) [26,27]. According to the size of PrP^Sc^ fragments seen in Western blot analysis two strains of prions are classified: type 1 PrP^Sc^ and type 2 PrP^Sc^. The relative molecular mass of type 1 PrP^Sc^ is around 21 kDa while it is around 19 kDa for type 2 PrP^Sc^ [28].

**Figure 2:**
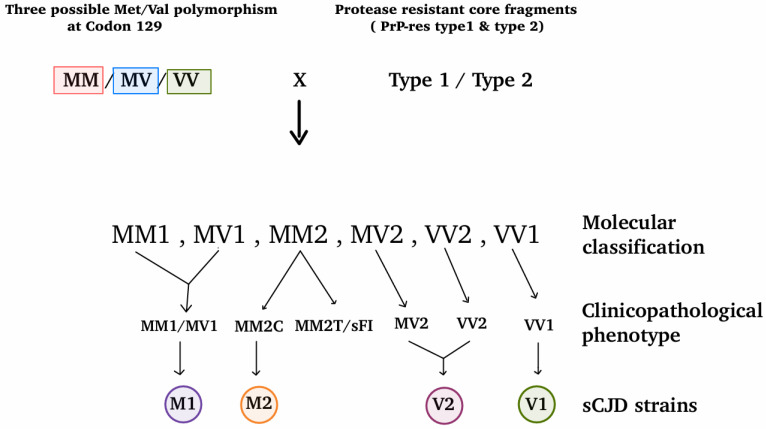
Diagrammatic representation of the molecular, clinicopathological and associated prion strains of sCJD.

In sCJD, clinicopathological manifestations are influenced by several factors such as PRNP polymorphism at codon 129 (involving Methionine and Valine) and types of prion strains. These combinations lead to classification of sCJD into six molecular subtypes namely: MM1 (Met/Met and PrP type 1), MM2 (Met/Met and PrP type 2), MV1 (Met/Val and PrP type 1), MV2 (Met/Val and PrP type 2), VV1 (Val/Val and PrP type 1) and VV2 (Val/Val and PrP type 2) [24,28]. The sCJD MM1 and MV1 are combined into a single subgroup (sometimes referred to as MM(V)1) as they exhibit common clinicopathological features [29,30]. Furthermore, on the basis of clinicopathological phenotypes the MM2 type sCJD is further classified into MM2-cortical-type (MM2C) and MM2-thalamic-type (MM2T) [19]. However, the clinical and neuropathological similarity to Fatal familial insomnia (FFI), leads to thalamic cases (MM2T) reclassified as sFI. Consequently, this classification results in the so-called “histotype” or five subtypes of sCJD comprising, MM1/MV1, MM2C, MV2, VV1 and VV2. Experimental transmission studies confirm different strains of sCJD for these clinicopathological subtypes. These include four strains namely; M1 (sCJD MM1/MV1 subtypes), M2 (sCJD MM2C subtype), V1 (sCJD VV1 subtype), V2 (sCJD VV2 and MV2 subtypes) [26]. Figure 2 shows diagrammatic representation of the molecular, clinicopathological and associated prion strains of sCJD. Interestingly, 14-3-3 protein is Negative in CSF analysis of MM2 thalamic type while its positive for others [8]. The clinicopathological manifestations (age of onset, clinical presentation, disease duration) differ in each subtypes. The classification system described by Piero Parchi and Pierluigi Gambettii is vastly followed by the majority of Prion field. Patients who are homozygous for methionine at codon 129 of the PRNP gene have an increased susceptibility to sCJD and represent the most common genotype observed in affected individuals [8,31].

Diagrammatic representation of the molecular, clinicopathological and associated prion strains of sCJD. The three possible methionine/valine polymorphism at codon 129 of *PRNP* (MM/MV/VV) are shown along with two protease-resistant core fragments (PrP^res^ type 1 and type 2). By pairing the *PRNP* codon 129 genotype with the two distinct PrP^res^ types results in six molecular classifications of sCJD (MM1, MV1, MM2, MV2, VV2, VV1).

Subsequent classification of sCJD by variations in the clinical and pathological phenotypes are MM1/MV1, MM2C, MM2T/sFI, MV2, VV2, VV1. Distinct strains of sCJD are M1, M2, V2, V1.

MM (Met/Met), MV (Met/Val), VV (Val/Val), MM1 (Met/Met and PrP type 1), MM2 (Met/Met and PrP type 2), MV1 (Met/Val and PrP type 1), MV2 (Met/Val and PrP type 2), VV1 (Val/Val and PrP type 1), VV2 (Val/Val and PrP type 2), MM2-cortical-type (MM2C), MM2-thalamic-type (MM2T), Sporadic Fatal Insomnia (sFI), M1 (sCJD MM1/MV1 subtypes), M2 (sCJD MM2C subtype), V1 (sCJD VV1 subtype), V2 (sCJD VV2 and MV2 subtypes).

### 4.2. Inherited CJD/Familial CJD [fCJD]

This form of CJD is rare, making up about 10–15% of all CJD cases [11,21]. fCJD (Table 2) occurs due to inherited changes in the prion protein gene (PRNP) on the chromosome 20p13 (short arm band 13) in Autosomal dominant way [32]. The E200K point mutation in the PRNP gene is the most frequent cause of fCJD detected worldwide. It accounts for more than seventy percent of affected families with a high prevalence in countries such as Chile, Italy, Slovakia, and Jewish communities of Libya and Tunisia [33,34]. The D178N mutation is also associated with fCJD and usually leads to a slower onset compared to sCJD. Specifically, the Codon 129V (Valine)D178 haplotype mutant allele is associated with fCJD while the 129M (Methionine)D178 haplotype is linked to FFI (Fatal familial insomnia) [27]. Symptoms like progressive memory loss, behavioral disturbance, ataxia, dysarthria, and myoclonus are experienced by the affected individuals. The age of onset in CJD varies depending on the subtype. SCJD, the most common form, typically presents in the sixth decade of life, whereas variant CJD tends to affect younger individuals, often between 30 and 50 years of age. Familial forms may also present earlier, depending on the underlying genetic mutation [32,35].

### 4.3. Acquired CJD/Iatrogenic CJD (iCJD)

Iatrogenic CJD (Table 2) is very rare because of improved and better medical practices and accounts for less than 1% of all cases [11,18]. The main cause is likely due to exposure of contaminated medical instruments or tissues from an infected person. The iCJD infection via infected corneal transplant was the first documented case and was described by Duffy in 1974 [36,37]. This can occur through contaminated surgical tools, blood transfusions from infected individuals, gonadotrophic hormone treatments, human-derived pituitary growth hormone, or corneal and meningeal grafts [20]. Since the use of human-derived hormones was discontinued, it can be considered that it is no longer a risk for these types of treatments. The incubation period can last from 1 to 42 years with symptoms similar to those of sCJD, including rapid cognitive decline, myoclonus, and ataxia [38]. However, the onset timing can vary based on how the victim was exposed. Since 1985, synthetic versions of human growth hormone have been used, removing this specific risk. Iatrogenic CJD can also develop if surgical tools used during brain operations on a CJD patient are not properly cleaned and sterilized. Prions are resistant to conventional methods of decontamination/sterilization; hence the presence of contaminated instruments can be a serious risk not only to the patients but also the healthcare providers. So, in the case of absence of strong evidence against the presence of prion disease in a neurosurgical patient, it is advised to take precautionary measures to prevent iatrogenic transmission of the disease via surgical instruments, as the neural tissue has the highest infectious burden for prion diseases [36]. A case study by Sharma et al., 2022, emphasizes the risk of misdiagnosis even in patients who underwent kidney transplantation, where CJD can be masked by neurological complications post-surgery [39]. Nevertheless, increased awareness of these risks has greatly plunked down the cases of iatrogenic CJD [3,36,40].

### 4.4. Variant CJD

Variant CJD (Table 2) is linked to bovine spongiform encephalopathy (BSE), commonly known as “mad cow disease.” It became a public health concern in the mid-1990s which also accounts for less than 1% of all CJD cases [3,11,18]. The primary cause is consuming contaminated beef products from the cattle suffering from BSE [23,41]. Following the discovery of the link between BSE and vCJD in 1996, the introduction and implementation of strict regulations on beef consumption and animal feed significantly reduced the risk. Unlike the other types, vCJD affects people in their third decade of life [41]. The disease usually starts with psychiatric symptoms like depression, delusions, anxiety, and changes in behavior and are the initial phase of the disease lasting around six months [24,41]. This is followed by neurological signs such as ataxia, confusion, involuntary movements, and myoclonus. The condition often progresses more slowly than sCJD, potentially lasting over a year with the average survival of 14 months from the disease onset [24]. The average time for the onset of variant CJD symptoms after initial infection, known as the incubation period, is still unclear [3]. Surprisingly, PSWCs (periodic sharp wave pattern) during EEG is usually not seen except for terminal stages of the disease [24,41]. 14-3-3 in CSF is only positive in 50% of cases. The 14-3-3 protein is widely used as a supportive biomarker in the diagnosis of CJD. However, it does not play a causal role in disease pathogenesis. Instead, its presence in cerebrospinal fluid reflects rapid neuronal damage and cell death. Elevated levels of 14-3-3 protein are therefore considered a nonspecific indicator of acute neurodegeneration and may also be observed in other neurological conditions.

The “hockey stick sign” in the pulvinar regions of thalamus during MRI brain scan (Fluid-attenuated inversion recovery (FLAIR) and diffusion-weighted imaging (DWI)) is present in ninety percent of the cases [41]. Tonsil biopsy can be used to check for presence/deposition of type2B or type4 PrP^res^ in most of the cases; particular for vCJD [24]. The BSE/vCJD PrP^res^ is referred to as type 2B and type 4 which accumulates in the peripheral tissues in clinical vCJD [41]. Still, a definitive diagnosis is based on brain biopsy usually post-mortem as other types of CJD. vCJD occurring in young adults and its susceptibility associated with the PRNP polymorphism at the codon 129, is 129Met/Met in almost all clinical cases [42]. However, other genotypes (Met/Val, Val/Val) may also be susceptible with long asymptomatic incubation period. Tissues containing abnormal PrP which can be infectious in clinical (129 Met/Met) type of vCJD include frontal cortex, pituitary gland, cervical lymph node, tonsil, appendix, distal ileum, spleen, thymus, lungs, heart, liver, kidney, salivary gland, pancreas, thyroid, adrenal gland, bone marrow, skeletal muscle, optic nerve, testis, ovary, rectum, retina, skin, uterus and blood plasma. Some of these tissues are also involved in subclinical types of vCJD (129Met/Met and 129Met/Val), however most of them have no data [42]. vCJD can be transmitted via blood [41,42,43,44]. Unlike sCJD the PrP^Sc^ is widely distributed in the lymphoreticular system of patients with vCJD [45,46,47]. The blood transmission related vCJD are linked only to recipient receiving non-leucoreduced blood [48,49,50,51]. The median age at death is 28 years [19].

### 4.5. Classification According to Clinical Presentation

Before the molecular and phenotypic analysis-based classifications, CJD was classified according to clinical presentation namely, 1. Classic type, 2. Heidenhain variant, 3. Brownwell-Oppenheimer variant, 4. Thalamic degeneration and 5. Amyotrophic form. The “classic triad” (rapidly progressive cognitive dysfunction, myoclonus and EEG showing PSWCs) was traditionally called as the Classic type or Myoclonic type. Akinetic mutism is seen within months after the onset of symptoms. The Heidenhain variant is characterized when there are visual symptoms like visus defigurata/visual agnosia (can see objects but cannot recognize or interpret them correctly), Achromatopsia/Dyschromatopsia/Defective color vision, Cortical Blindness and Visual Anosognosia/Anton’s Syndrome (cortically blind patient denies being blind and confabulates visual descriptions). PSWCs on EEG at occipital lobe, acute cognitive dysfunction and other neuropsychiatric symptoms are observed. The ataxic variant also known as Brownwell-Oppenheimer variant starting with dementia, and cerebellar symptoms with Negative PSWCs on EEG are typical. Sporadic Fatal Insomnia, another name for Thalamic degeneration, presents with psychiatric symptoms, autonomic dysfunction and obvious sleep disorders. Basic rhythm instead of PSWCs are present in EEG and myoclonus is inconspicuous in this type. The last type, Amyotrophic form is uncommon and is distinguished by remarkable muscle atrophy [8].

The symptoms of CJD happen because of the slow decay of nerve cells in the brain, which relates to the buildup of abnormal prion proteins. A histopathological exam usually shows vacuolation from neuronal loss, leading to a sponge-like texture in the brain. Some areas of the brain may look spongy due to the prion infection affecting those regions [52]. In advanced stages, the disease resembles other prion diseases making the diagnosis challenging [23]. Diseases such as Immune-mediated encephalitis, N-methyl-D-aspartate receptor antibody encephalitis, Voltage-gated potassium channel complex antibody encephalitis, AMPA receptor antibody encephalitis, GAB*A_A_*/*GABA_B_R* antibody limbic encephalitis, glycine receptor antibodies, Hashimoto’s encephalitis, progressive multifocal encephalopathy, Subacute sclerosing panencephalitis, Toxic metabolic syndromes such as Wernicke’s encephalopathy, Neoplastic conditions, Paraneoplastic conditions, Large vessel stroke, Dural arteriovenous fistula, Posterior reversible encephalopathy syndrome, Primary CNS vasculitis, Alzheimer’s, Dementia with Lewy bodies and Huntington’s diseases can mimic CJD and are potential differential diagnosis making the final diagnosis harder [23,53].

## 5. Histopathology and Pathophysiology

**Figure 3:**
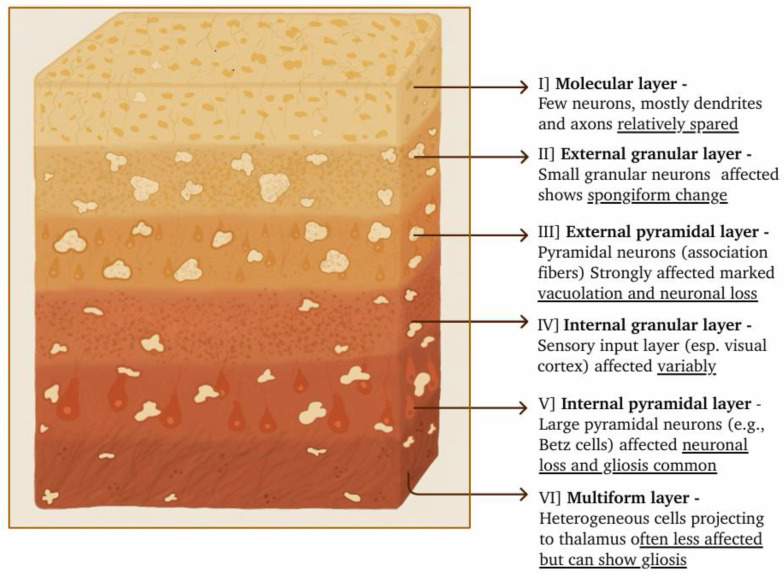
An image illustrating spongiform vacuolation in different layers of the Brain cortex.

The pathological hallmark of CJD is the accumulation of abnormally folded prion proteins (PrP^Sc^) (Figure 3) in the brain. The cerebral cortex demonstrates a characteristic pattern of selective vulnerability in prion disease. The molecular layer (Layer I), mainly composed of dendritic and axonal processes with few neuronal cell bodies, is relatively spared. The external granular layer (Layer II), containing small granular neurons are affected and shows spongiform change. The external pyramidal layer (Layer III), which contains pyramidal neurons involved in cortico-cortical association pathways [association fibers], is strongly affected, exhibiting marked vacuolation and neuronal loss. The internal granular layer (Layer IV), a recipient of sensory input (especially in the visual cortex), is affected variably. The internal pyramidal layer (Layer V), containing large pyramidal neurons such as Betz cells, demonstrates neuronal loss and gliosis. The multiform layer (Layer VI), containing heterogeneous neurons projecting to the thalamus, is less affected, although gliosis may be present.

Figure 3 shows spongiform vacuolation in different layers of the cortex. Some individuals can be found with amyloid plaques [19]. The spread of prions throughout the brain accelerates the progression of the disease. Misfolded prions can invade various areas, including the cortex, cerebellum, and basal ganglia [54]. As a result, regions of the brain that control thinking, movement, and memory become severely affected. Recent advances in structural biology have significantly expanded our understanding of prion architecture, particularly through the use of cryo-electron microscopy. This allows for the visualization of infectious prion fibrils with near-atomic resolution. The golden standard for diagnosing prion diseases, including CJD, is the detection of protease-resistant PrP^Sc^ in the neural tissue [55]. These misfolded proteins propagate by prompting normal prion proteins (PrP^C^) to assume aberrant confirmation, thereby initiating a cascade of protein misfolding [19].

**Figure 4:**
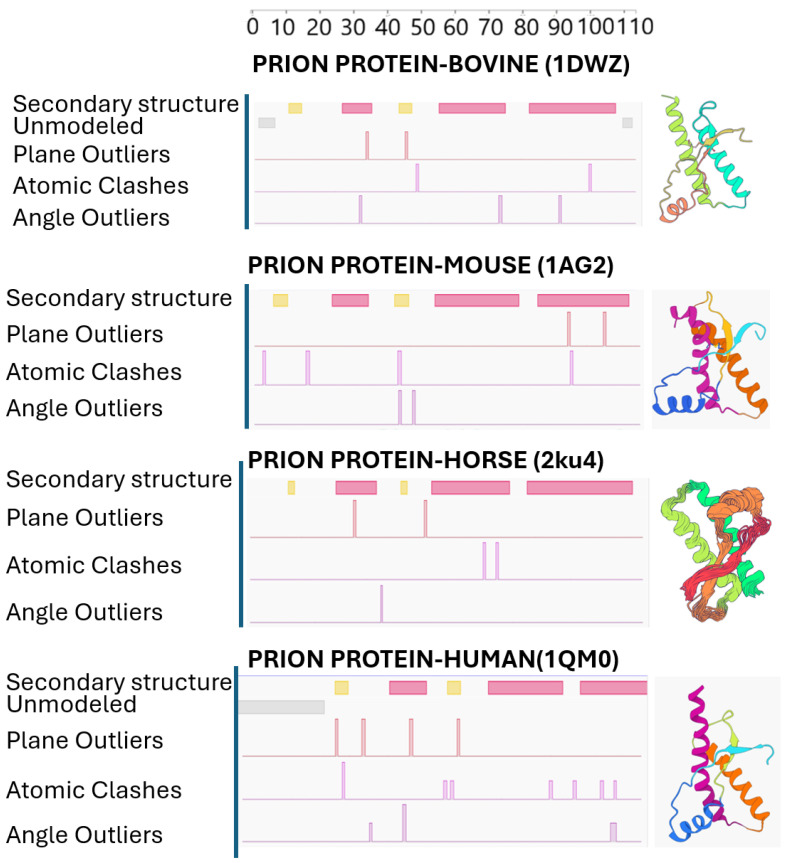
Comparative structural analysis of prion proteins from different species. Secondary structure elements and structural validation metrics are shown for bovine (1DWZ), mouse (1AG2), horse (2ku4), and human (1QM0) prion proteins. The diagrams illustrate α-helical and β-sheet regions along the amino acid sequence, together with quality assessment parameters including unmodeled regions, plane outliers, atomic clashes, and angle outliers. Representative three-dimensional structures are presented on the right.

Prion proteins play a vital role in this process. Normally, PrP^C^ is present in brain cells; however, in CJD, this protein misfolds into a beta-sheet-rich form known as PrP^Sc^, a variant which is resistant to breakdown by proteolytic enzymes (such as PK) [56]. The buildup of PrP^Sc^ in neurons causes cell death and problems with synapses and also leads to the typical sponge-like changes in brain tissue, which can be seen through histopathological analysis [13,20]. As misfolded prions accumulate, neurodegeneration occurs causing serious damage to both neurons and glial cells. The process results in the formation of vacuoles in neurons, giving the affected brain tissue its characteristic appearance. Primarily in gray matter, Astrocytic gliosis or fibrous proliferation of the astrocytes are observed [39,54]. The middle and deep cortical laminae (layers 3 to 5) typically exhibit spongiform vacuolation. Deposition of prion protein are often found in superficial layers in vCJD while its deeper layers are often found in cases of sCJD [54]. Figure 4 shows the comparative structural validation analysis of prion proteins (NMR models). It shows that the horse structure (3KU4) has the lowest number of plane outliers, atomic clashes, and angle outliers, indicating high ideal geometry. However, the human structure (1QM0) exhibits the highest number of abovementioned deviations, suggesting greater local structural irregularity. Bovine (1DWZ) and rat (1AG2) proteins exhibit intermediate behavior. While these validation metrics primarily reflect structural quality, the trend is consistent with known differences in β2-α2 loop rigidity, where increased structural order in horse prion protein correlates with resistance to prion diseases, whereas increased flexibility in the human protein may facilitate pathogenic conformational changes. However, these metrics are influenced by structure resolution, refinement quality and different construct lengths. Despite overall structural similarity, subtle differences in secondary structure organization and local geometry may contribute to species-specific susceptibility to prion misfolding and disease propagation.

Importantly, studies such as those by Vázquez-Fernández et al. have shown that prion aggregates adopt highly ordered amyloid structures rich in β-sheets with an architecture of parallel, intermolecular β-sheets. These findings support the concept that prion propagation is based on a template conformation, in which misfolded PrPs imposes its structure on native PrPs [57].

Importantly, cryo-electron microscopy data also highlighted the structural heterogeneity of prion strains, suggesting that different PrPs conformations may account for the phenotypic variability observed in different forms of CJD. Despite these advances, limitations remain due to difficulties in determining the full-length structures of prion proteins and capturing early, misfolded intermediates. Nevertheless, cryo-electron microscopy represents a breakthrough in prion research and provides a foundation for structure-based therapy design [57].

## 6. Diagnostic Approaches

A healthcare provider, typically a neurologist, uses clinical diagnosis, laboratory tests, imaging studies, biopsy and post-mortem examination to diagnose the disease.

Clinical diagnosis includes a detailed neurological examination (including the onset and progression of symptoms), history (in suspected cases of familial CJD, family history may be relevant) and symptoms. During neurological examination, the presence of myoclonus, ataxia and rapidly progressive cognitive decline raises the concern of CJD.

Cerebrospinal fluid (CSF) analysis and real-time quaking-induced conversion (RT-QuIC) are used for laboratory tests. The detection of biomarkers such as 14-3-3 proteins and Tau (total tau/t-tau) proteins in the CSF are indicative of CJD, which can be detected using Enzyme Linked Immunosorbent Assay (ELISA) [58,59,60,61]. However, they may be found in other neurodegenerative diseases. The characteristic neuropathological hallmark of TSE is the presence of PrP^Sc^, although numerous methods have been developed to detect PrP^Sc^; the presence of only minute/scant quantities in bodily fluid like CSF and blood, coupled with the absence of specific antibodies, has made accurate differentiation between PrP^Sc^ from PrP^C^ challenging [62]. RT-QuIC is a highly sensitive next-generation test (sensitivity of 90–100% and specificity of 95–100%) which can detect 14-3-3 and Tau proteins in samples such as CSF, saliva, olfactory mucosa and urine [63,64]. It involves the use of rPrP/recombinant PrP (substrate) and CSF containing PrP^Sc^, which then is induced to aggregate. Thioflavin T (fluorescent dye) subsequently binds to the aggregated PrP ^Sc^, resulting in a change in the thioflavin T emission spectrum which then enables the reaction to be monitored in real time [65,66,67]. However, the test currently does not amplify PrP^Sc^ in cases of vCJD. Due to its importance in clinical utility, both the European Centre for Disease Prevention and Control as well as The United States of America’s Centers for Disease Control and Prevention (CDC) have incorporated RT-QuIC into their diagnostic criteria for CJD [17,68].

**Figure 5:**
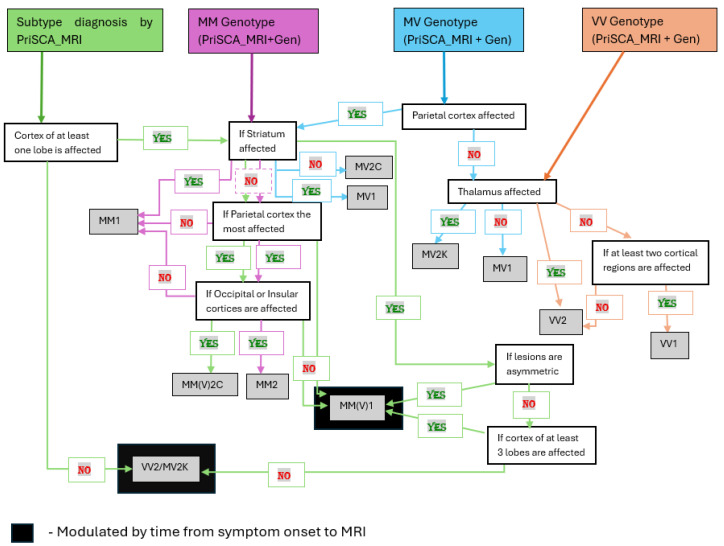
Diagnosis of sCJD subtypes with PriSCA_MRI and PriSCA_MRI + Gen. Green—subtype diagnosis by PriSCA_MRI, Violet—PriSCA_MRI + Gen (MM genotype), Blue—PriSCA_MRI +Gen (MV genotype), Orange—PriSCA_MRI +Gen (VV genotype). The color applies to respective boxes and arrows. The queries in the white boxes outline the decision tree and lead to either the subtype diagnosis in the gray boxes or to additional queries directed at further defining MRI lesions and diagnoses. PriSCA_MRI (MRI alone) and PriSCA_MRI + Gen (MRI with *PRNP*129), MM (Met/Met), MV (Met/Val), VV (Val/Val), MM1 (Met/Met and PrP type 1), MM(V)2C (Met/Met or Met/Val, PrP type 2 and cortical subtype), MM2 (Met/Met and PrP type 2), VV2 (Val/Val and PrP type 2), MV2K (Met/Val, PrP type 2 and kuru plaques subtype), MM(V)1 (MM1 and MV1), MV2C (Met/Val, PrP type 2 and cortical subtype), MV1 (Met/Val and PrP type 1), VV1 (Val/Val and PrP type 1), MRI-Magnetic Resonance Imaging.

Imaging studies include Magnetic Resonance Imaging (MRI) and Electroencephalography (EEG). MRI, especially diffusion-weighted imaging (DWI) revealing the hyperintensities in thalamus, cortical areas and basal ganglia, can suggest CJD but are not definitive. DWI is highly sensitive at detecting early cortical and sub cortical changes [18]. vCJD shows the characteristic “Pulvinar sign” which is the presence of symmetrical hyperintensity in the posterior thalamus, relative to the anterior putamen, on the T2-weighted or FLAIR MRI [18,69]. Bizzi A. et al., 2021 [30] have designed a procedure for sCJD subtype diagnosis using Diffusion MRI. It includes two procedures, namely PriSCA_MRI (MRI alone) and PriSCA_MRI + Gen (MRI with PRNP129). PriSCA_MRI (Figure 5) stands for prion subtype classification algorithm with MRI.

1. *Subtype diagnosis by Diffusion MRI alone which can determine MM(V)1, MM(V)2C and VV2/MV2K is as follows:* the final diagnosis begins by assessing whether the cortex of at least one lobe of the brain is affected. If NOT affected, the diagnosis is VV2/MV2K (positive predictive value around 91%), which is influenced by the time from onset of symptoms to MRI. If the cortex is affected the next step is to ascertain whether the Striatum is affected. When it is NOT affected the analysis focuses on cortical distribution. The diagnosis of MM(V)C is concluded when the parietal cortex is the most affected region and involvement of occipital or insular cortices (positive predictive value around 60%). The outcome is MM(V)1 if the parietal cortex is NOT the most affected region (positive predictive value around 67%), which is again modulated by the time from symptom onset to MRI. If the striatum is affected, the analysis focuses on lesion symmetry. MM(V)1 is diagnosed again when there are Asymmetric lesions (positive predictive value around 81%) modulated by the time from symptom onset to MRI. If the lesions are symmetric, the next criterion is to determine whether at least three cortical lobes are affected. If three or more lobes are involved, it can be determined as VV2/MV2K (positive predictive value around 74%). If less than three lobes are involved it results in MM(V)1 (positive predictive value around 46%) modulated once again by the time from symptom onset to MRI [30].

*2. Diagnosis of sCJD subtypes with PriSCA_MRI + Gen is as follows*: 1. MM genotype (Figure 5). The decision begins by determining whether the striatum is affected and MM1 subtype is diagnosed when the striatum is involved (positive predictive value around 98%). If the striatum is not involved the next criterion is whether the parietal cortex is the most affected region. If it is NOT, then the diagnosis is again MM1 (positive predictive value around 83%). If the parietal cortex is the most affected region, the next step is to assess if the insula or occipital cortex are involved. If neither of them are affected, then the diagnosis is once again MM1 (positive predictive value around 81%). However, it is determined as MM2 subtype if it involves insular or occipital cortices (positive predictive value around 60%). 2. MV genotype (Figure 5); the classification begins by assessing if the parietal cortex is affected. If YES, the next step is to determine the involvement of striatum. MV1 is diagnosed when there is striatum involvement (positive predictive value around 59%). However, it is concluded as MV2C subtype if the striatum is not affected (positive predictive value around 67%). If there is no involvement of parietal cortex, the next step is to analyze whether the thalamus is affected. Absence of thalamic involvement leads to diagnosis of MV1 again (57%). Whereas the involvement of the thalamus results in MV2K subtype (positive predictive value around 86%). 3. VV genotype (Figure 5); for VV genotype the first criterion is to point out if the thalamus is affected. Involvement of thalamus results in the diagnosis of VV2 Subtype (positive predictive value around 100%). Absence of thalamic involvement leads to assessment of whether at least two cortical regions are affected. If YES, the diagnosis is VV1 subtype (positive predictive value around 79%). Involvement of less than two cortical regions is determined again as VV2 subtype (positive predictive value around 95%) [30]. The positive predictive value is obtained by Bizzi A. et al., from diagnosing the number of patients involved in their study.

The five sCJD subtypes classified by this method are MM(V)1, MM(V)2C, VV1, VV2, MV2K. This may lead to early diagnosis in turn leading to prolonged survival of the patient.

Typical practice include 1. Initial MRI at presentation, 2. Repeat MRI if Diagnosis uncertain or Initial MRI normal or Clinical progression continues. Cortical involvement (“cortical ribboning”) is typically earliest which may occur within days to weeks of symptom onset. Some patients may show isolated cortical changes initially which correspond clinically to Cognitive decline and Behavioral changes. Striatal involvement appears later. Paolo V et al. 2019 [70], did imaging at multi-month intervals (4, 6, 8, 9, 11, 13 and 17 months). The said case study shows cortical involvement from first scan which was stable till 11 months and the striatal involvement appeared around the 11th to 13th month (left striatum at month 11 and bilateral involvement of anterior striatum at month 13) [70].

Presence of Periodic sharp wave complex (PSWC) pattern on EEG is a strong indicator of prion disease but not always present in every case [71,72]. In sCJD the PSWC can be simple sharp waves (biphasic and triphasic) or complex (mixed spikes, slower waves and polyspikes) which recurs every 0.5–2 s. The typical duration of waves ranges from 100 to 600 ms [71]. Occurrence of lateralized PSWC also known as PLED, can indicate the early stage of sCJD [44,71]. It should be taken into note that PSWC can be influenced by external stimulation such as sedative medicines (such as midazolam, diazepam) [59,71]. FRIDA-like EEG patterns can be found in early stages of sCJD which are nonspecific EEG findings [71]. The EEG in iCJD resembles that of sCJD, whereas in fCJD patients with mutation in 178, 200, 210 and without myoclonus, the EEG does not exhibit PSWC. In vCJD patients the PSWC is absent, which is also one of the diagnostic criteria for this particular subtype [71]. If the diagnosis remains doubtful, a brain biopsy or autopsy is required to check the presence of spongiform changes and accumulation of prion protein [73].

In biochemical characterization of CJD, the “Gold standard” is Western blotting of brain tissue as well as genotyping of codon 129 located in chromosome 20 encoding for prion gene [74]. Tonsil biopsy or adenoid biopsy can be done particularly in variant CJD to find abnormal prions [69,75]. A prototype blood and urine test for variant CJD has also been developed. Detection of PrP^Sc^ in the CSF, BLOOD (Sensitivity Of 100%) and urine samples (Sensitivity Of 93%) of patients with vCJD is done using Protein Misfolding Cyclic Amplification (PMCA) [76]. Genetic testing to find the presence of mutation in the gene producing normal protein may indicate familial CJD if positive [77]. Detection and characterization of prion proteins from patients suffering from CJD has been developed by using a capillary-based Western assay using JESS Simple Western/ProteinSimple by Myskiw J. et al., 2023 which reduces the misdiagnosis using standard Western blotting protocols [74]. Neurofilament light chain (NF-L) is an accessible and valuable marker of neurological injury/neurodegeneration which can be detected in both CSF and Blood [78]. Their levels are elevated in cases of axonal injury and nerve cell degradation [79,80]. Neurofilaments which are found in the central nervous system and the Ganglia of Peripheral Nervous System are the key components of Cytoskeleton in axons and dendrites. Out of four subunits of Neurofilaments (NF-H, NF-M, NF-L and α-Internexin), the NF-L/Neurofilament light chain is a promising biomarker due to its abundance and solubility [79]. However, there is no correlation between the NF-L values and the duration of the disease [79]. In cases of sCJD it has sensitivity ranging from 90 to 96% with specificity of 80–85%. α-Synuclein is a Biomarker which helps in differentiating CJD from Lewy Body dementia and Parkinson’s disease [22]. Neuron-Specific Enolase (NSE) which can be found in blood and CSF is elevated in neurodegenerative diseases and their levels increases as CJD progresses [22]. FDG-PET (Positron emission tomography using fluoro-2-deoxy-D-glucose as tracer) can be used in cases of sCJD where it detects reduced glucose metabolism in the cortical regions [69].

The United States of America’s Centers for Disease Control and Prevention (CDC) defines the following diagnostic criteria (Table 3):

(a) sCJD: *Definite sCJD*: when diagnosed either together or alone by standard neuropathological with Western bolt confirmed protease-resistant PrP, immunocytochemical, and presence of scrapie-associated fibrils [69]. *Probable sCJD*: when there is neuropsychiatric disorder with RT-QuIC positive from CSF or different tissues, or dementia which is progressing rapidly and with at least two out of four confirmed clinical features namely myoclonus, visual/cerebellar signs, akinetic mutism and pyramidal/extrapyramidal signs [69]. As for laboratory tests, positive results in one of tests including EEG, 14-3-3 CSF assay, DWI/FLAIR (MRI) high signal in putamen/caudate/cortical regions within 2 years of disease duration. *Possible sCJD:* is confirmed when at least two of the previously mentioned clinical features are present, accompanied by progressive dementia, negative test results with illness lasting less than two years and without routine investigations [68].
(b) Iatrogenic Creutzfeldt–Jakob disease (iCJD) is diagnosed when a recipient of human cadaveric-derived pituitary hormone is experiencing progressive cerebellar syndrome or sCJD with a recognized exposure of risk, e.g., antecedent neurosurgery with dura mater implantation [69].

Familial Creutzfeldt–Jakob disease (fCJD) is considered when definite or probable CJD patient has, again, definite or probable CJD in a first-degree relative and/or neuropsychiatric disorder, and disease-specific *PrP* gene mutation [69]. The sole method to confirm a diagnosis of CJD is to examine the brain tissue by conducting a brain biopsy or post-mortem examination of the brain.

Criteria for clinical diagnosis of CJD.

## 7. Management and Prognosis

There is currently no proven cure for Creutzfeldt–Jakob disease (CJD), and treatment remains primarily supportive. Clinical management focuses on ensuring patient comfort and alleviating symptoms through pharmacological and non-pharmacological approaches [81]. Despite ongoing clinical research, all forms of CJD are invariably fatal, with prognosis varying depending on the subtype.

SCJD typically leads to death within 6–12 months of symptom onset, whereas familial CJD (fCJD) may follow a slightly longer clinical course. Variant CJD (vCJD) often progresses more slowly, with disease duration exceeding one year in some cases. Importantly, early diagnosis does not alter disease progression but may improve patient management and planning of palliative care [81,82].

As the disease progresses, patients require comprehensive supportive care, including assistance with feeding, mobility, hygiene, and daily activities. Dysphagia (difficulty swallowing) and reduced nutritional intake are common in advanced stages, often necessitating enteral feeding via nasogastric or percutaneous endoscopic gastrostomy tubes [83,84,85,86,87]. Urinary catheterization is also frequently required.

Symptom management plays a central role in improving quality of life. Myoclonus and seizures may be treated with agents such as clonazepam or valproate. Antidepressants and antipsychotics may be administered to alleviate psychiatric symptoms, including anxiety and depression [81].

Nutritional management is another critical component of care. Malnutrition may result from dysphagia or decreased appetite, and appropriate nutritional support is essential. Current recommendations include monitoring of swallowing function, individualized dietary planning, and supplementation where necessary. In advanced cases, long-term enteral feeding may be required [84].

Given the rapid progression and fatal nature of CJD, palliative and end-of-life care are essential. Management strategies focus on symptom relief, maintaining patient dignity, and providing psychological and emotional support to both patients and caregivers.

## 8. Emerging Research and Future Perspectives

CJD remains one of the most challenging neurodegenerative diseases due to its rapid progression, mortality, and lack of effective disease-modifying therapies. Despite significant advances in diagnostic techniques, early and accurate detection remains challenging, especially in the early stages. Methods such as real-time tremor-induced conversion (RT-QuIC) [88] and advanced neuroimaging have significantly improved diagnostic sensitivity and specificity; however, their availability and implementation in routine clinical practice remain limited.

Recent advances in molecular diagnostics also point to the potential role of sequencing-based and liquid biopsy-based methods in detecting prion diseases. Although still in the early stages of development, these techniques aim to identify prion-associated biomarkers in readily available biological fluids, such as blood and cerebrospinal fluid. Highly sensitive assays combined with next-generation sequencing (NGS) may enable the detection of trace amounts of changes in gene expression or the identification of prion-associated genetic predispositions (e.g., mutations in the PRNP gene) or protein signatures. Liquid biopsy-based methods have the advantage of being minimally invasive and may allow for repeated monitoring of disease progression. However, they rely on indirect detection methods. Therefore, further research is needed to validate these methods and their clinical utility [89].

Recent advances in prion research have focused on both therapeutic development and a deeper understanding of disease mechanisms at the molecular and cellular levels. A growing body of scientific evidence is contributing to the elucidation of the processes underlying prion formation, aggregation, and propagation in the central nervous system [78]. These mechanistic insights provide a crucial foundation for the development of targeted therapeutic strategies and improved disease models. From a structural perspective, techniques such as cryo-electron microscopy and computational methods, including molecular dynamics (MD) simulations, have significantly advanced our understanding of prion protein architecture and conformational dynamics [90]. In the absence of a complete, high-resolution structure of PrP^Sc^, MD simulations provide valuable information on protein stability, folding transitions, and the influence of environmental factors such as pH, temperature, and mutations. These studies suggest that specific regions of PrP^C^, particularly the α-helical domains, may exhibit intrinsic instability and convert to β-pleated sheet-like conformations under certain conditions, supporting the hypothesis that subtle structural perturbations can initiate misfolding processes [91]. However, computational models are based on simplified systems and may not fully capture the complexities of prion behavior in vivo. Experimental models have also evolved significantly. Three-dimensional brain organoids and transgenic animal models are increasingly being used to study prion biology and evaluate potential therapeutic compounds. For example, the expression of mutant human prion proteins in model organisms such as *Caenorhabditis elegans* has enabled high-throughput drug screening [92]. However, despite their usefulness, these models do not fully reflect the complexity of human prion diseases, limiting their predictive value for clinical outcomes. Based on these advances, several therapeutic strategies are currently being explored. Immunotherapeutic approaches, including monoclonal antibodies such as PRN100 and peptide- or dendritic cell-based vaccines, have shown promising results in preclinical studies [93,94]. Antisense oligonucleotide (ASO) therapy is another emerging strategy aimed at reducing prion protein expression by targeting PRNP mRNA, thereby limiting the availability of substrate for misfolding [95]. Although preclinical data suggest delayed disease progression, the long-term safety and clinical efficacy of these approaches remain to be confirmed.

Antisense oligonucleotide (ASO) therapy is another emerging strategy aimed at reducing prion protein expression by targeting PRNP mRNA, thereby limiting the availability of substrate for misfolding [95]. Although preclinical data suggest delayed disease progression, the long-term safety and clinical efficacy of these approaches remain to be confirmed.

**Figure 6:**
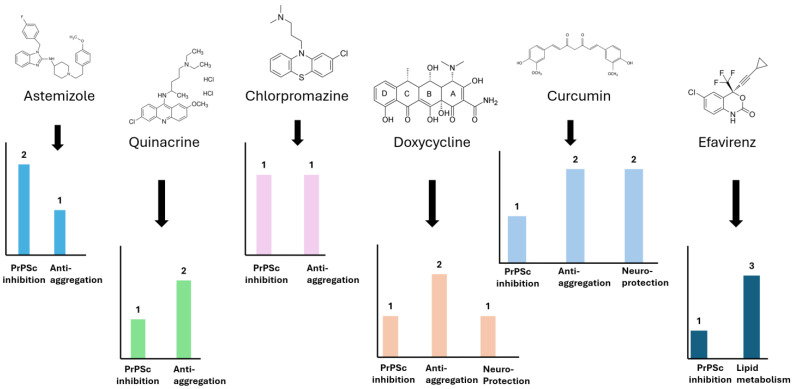
Molecular structures and qualitative comparison of selected therapeutic strategies in prion diseases. The histogram presents a semi-quantitative assessment of the primary molecular effects of selected compounds, including inhibition of PrP^Sc^ formation, reduction of PrP expression, anti-aggregation effects, neuroprotection, modulation of lipid metabolism, and immunomodulation. The scoring (0–3) is based on available preclinical and clinical evidence reported in the literature [58,93,96,97,98,99,100,101]. 1—weak/intermediate effect; 2—moderate (robust preclinical data); 3—strong (consistent data, including early clinical data).

Furthermore, several repurposed drugs (Figure 6)—including quinacrine [96,97,98], chlorpromazine [96], doxycycline [99,100], and efavirenz [58]—have demonstrated antiprion activity in experimental models.

Quinacrine [96,97,98] and chlorpromazine [96] have shown the ability to reduce prion accumulation in experimental models, although clinical efficacy remains limited. Doxycycline has demonstrated anti-aggregation properties and modest effects on survival, particularly when administered in early disease stages [99,100]. Efavirenz has been shown to reduce PrP^Sc^ levels through activation of cholesterol metabolism pathways; however, its effectiveness in human prion diseases has not been conclusively demonstrated [58].

Importantly, many of these compounds have failed to show significant benefit in clinical settings, highlighting the gap between experimental efficacy and clinical applicability (Table 4).

Emerging evidence suggests that certain bioactive compounds, including flavonoids (e.g., quercetin, chrysin, naringenin), anthocyanins, and curcumin, may exert neuroprotective effects through mechanisms such as antioxidant activity and modulation of signaling pathways [101] (e.g., Phosphoinositide 3-kinase (PI3K)/ Protein kinase B (Akt) and Keap1–Nrf2). However, these approaches remain experimental and lack robust clinical validation.

Consequently, supportive care remains the cornerstone of CJD treatment. Nutritional support is particularly important because patients often develop dysphagia, reduced oral intake, and progressive weight loss. Interventions such as enteral nutrition can improve patient comfort and quality of life, although they do not alter disease progression. New research is also examining potential links between prion diseases and systemic factors, including changes in gut microbiota and viral infections such as SARS-CoV-2 [102]. While these findings are intriguing, current evidence remains preliminary and insufficient to establish cause-and-effect relationships.

Despite significant progress in understanding prion biology, no therapy has yet demonstrated clear clinical efficacy. A major challenge remains translating promising experimental results into effective treatments for patients. Future research should focus on filling this gap by developing therapeutic approaches.

## 9. Conclusions

Prion diseases remain a significant scientific and clinical challenge. Although advances in understanding disease subtypes and diagnostic techniques have improved disease characterization, significant gaps remain in translating these discoveries into clinical benefit. Rapid disease progression, diagnostic complexity, and the lack of effective treatment are further challenges that require coordinated efforts in basic, clinical, and translational research. Early diagnosis and the development of effective therapeutic strategies remain within the reach of modern medicine.

## Figures and Tables

**Table 1:** PRNP gene mutations and their associated neuropathological phenotypes in inherited and suspected genetic prion diseases [17].

No.	Neuropathological Phenotype	Disease	PRNP MUTATIONS
1.	PLAQUE DOMINANT PHENOTYPE	Gerstmann–Sträussler–Scheinker Syndrome (GSS)	P102L, P105L, A117V, G131V, F198S, D202N, Q212P, Q217R, M232T, 192 bpi
2.	SPONGIFORM PHENOTYPE	CJD	D178N129V, V180I, V180I + M232R, T183A, T188A, E196K, E200K, V203I, R208H, V210I, E211Q, M232R, 96 bpi, 120 bpi, 144 bpi, 168 bpi, 48 bp del
3.	THALAMIC PHENOTYPE	Fatal familial insomnia (FFI)	D178N129M
4.	AMYLOID ANGIOPATHY/VASCULAR DEPOSITION	Vascular PRP amyloid	Y145s
5.	MIXED/ATYPICAL PHENOTYPE	Proven but unclassified prion disease	H187R, 216 bpi
6.	NO CONSISTENT PRION PATHOLOGY	Not proven prion disease but with neuropsychiatric disorder	I138M, G142S, Q160S, T188K, M232R, 24 bpi, 48 bpi, 48 bpi + nucleotide substitution in other octapeptides

**Table 2:** A comparative table highlighting the key differences between sporadic CJD (sCJD), familial CJD (fCJD), iatrogenic CJD (iCJD), and variant CJD (vCJD).

Feature	Sporadic CJD (sCJD)	Familial CJD (fCJD)	Iatrogenic CJD (iCJD)	Variant CJD (vCJD)
Etiology	Unknown (spontaneous PrP misfolding)	Autosomal dominant mutation in *PRNP* gene	Transmission via contaminated medical procedures	Exposure to bovine spongiform encephalopathy (BSE)
Proportion of cases	~85%	10–15%	<1%	<1%
Mean age of onset	~60–65 years	~50–60 years	~40–60 years (variable)	~25–30 years
Incubation period	Not applicable	Not applicable	Years to decades	Years to decades
Clinical presentation	Rapid cognitive decline, myoclonus, ataxia	Similar to sCJD, may vary by mutation	Similar to sCJD	Psychiatric symptoms, sensory disturbances, delayed neurological signs
Disease duration	4–6 months	6–18 months	14 months	~12–24 months
Median survival	~5–6 months	~12–18 months	Variable	~14 months
EEG findings	Periodic sharp wave complexes common	May be absent or atypical	Similar to sCJD	Usually absent
MRI findings	Cortical and basal ganglia hyperintensities	Variable	Similar to sCJD	“Pulvinar sign” (posterior thalamus)
CSF biomarkers	14-3-3, tau (elevated)	Variable	Similar to sCJD	Less consistent

**Table 3:** Diagnostic criteria for CJD adapted from CDC. sCJD cases are classified as definitive, probable, or possible based on a combination of clinical features, laboratory findings, and neuropathological confirmation. iCJD is diagnosed in cases of recognized exposure risk and fCJD is diagnosed in patients with CJD who have a first-degree relative with definite/probable CJD or neuropsychiatric disorder with mutation in the PrP gene.

SPORADIC CJD	IATROGENIC CJD	FAMILIAL CJD
*Definitive:* [II.e AND/OR II.f AND/OR II.g] + 1	6 OR sCJD with recognized exposure risk (such as dura matter implantation)	(Have definite or probable CJD) OR (definite or probable CJD in first-degree relative) AND/OR 2, 4, 7
*Probable:* [2 + II.d OR 3 + at least two of sections I.A-I.D] + [at least one of II.a to II.c)] AND 5		
*Possible:* [at least two of sections I.A-I.D] + 3 + [negative results of II (a to c) + within two years + 5]		

Legends: 1. Standard neuropathological examination. 2. Neuropsychiatric disorder. 3. Rapid progressive dementia. 4. Progressive neurological syndrome. 5. No routine investigations indicating an alternative diagnosis. 6. Progressive cerebellar syndrome in a recipient of human cadaveric-derived pituitary hormone. 7. Disease-specific PrP gene mutation. I.A-Myoclonus; I.B-Visual or cerebral signs; I.C-Akinetic mutism; I.D-Pyramidal or extrapyramidal signs; II.a-EEG with PSWCs; II.b-14-3-3 CSF assay within 2 years of disease duration; II.c-DWI/FLAIR (MRI) high signal in putamen/caudate/cortical regions (at least two of temporal, parietal, occipital); II.d-RT-QuIC positive from CSF or different tissues; II.e-Western bolt confirmed protease-resistant PrP; II.f-Immunocytochemical; II.g-Presence of scrapie-associated fibrils.

**Table 4:** Molecular mechanisms and therapeutic strategies targeting prion diseases.

Compound/Strategy	Molecular Target/Mechanism	Primary Molecular Effect	Stage of Development	Key Limitation
Astemizole	Interaction with PrP; modulation of lysosomal pathways	Reduction of PrP^Sc^ accumulation	Preclinical	Limited in vivo validation
PRN100 (monoclonal antibody)	Binding to PrP^C^, preventing pathological conversion	Inhibition of PrP^Sc^ formation	Early clinical	Limited clinical data
Immunotherapy (vaccines, dendritic cell-based)	Induction of immune response against prion proteins	Enhanced clearance of PrP/inhibition of propagation	Preclinical	Safety concerns; limited clinical validation
Antisense oligonucleotides (ASO therapy)	Targeting PRNP mRNA	Reduction of PrP expression	Preclinical/early clinical	Delivery challenges to CNS; unknown long-term effects
Quinacrine	Direct interaction with PrP^Sc^ aggregates	Inhibition of prion replication	Clinical (limited efficacy)	Poor clinical outcomes
Chlorpromazine	Membrane interaction; destabilization of PrP^Sc^	Reduction of PrP^Sc^ accumulation	Preclinical	Limited blood–brain barrier penetration
Doxycycline	Binding to amyloid fibrils	Anti-aggregation; fibril destabilization	Clinical trials	Modest efficacy
Efavirenz	Activation of CYP46A1 pathway	Modulation of cholesterol metabolism affecting PrP^Sc^	Preclinical	Indirect mechanism; unclear clinical relevance
Curcumin	Modulation of signaling pathways (e.g., PI3K/Akt, Keap1–Nrf2)	Neuroprotection; anti-aggregation	Preclinical	Poor stability and bioavailability

## Data Availability

The original contributions presented in this study are included in the article. Further inquiries can be directed to the corresponding authors.

## Abbreviations

Akt	Protein kinase B
ASO	Antisense oligonucleotide
BSE	Bovine spongiform encephalopathy
CDC	Centers for Disease Control and Prevention
CJD	Creutzfeldt–Jakob disease
CNS	Central nervous system
CSF	Cerebrospinal fluid
DWI	Diffusion-weighted imaging
ECDC	European Centre for Disease Prevention and Control
EEG	Electroencephalography
EFV	Efavirenz
FDA	Food and Drug Administration
FDG-PET	Fluorodeoxyglucose positron emission tomography
FLAIR	Fluid-attenuated inversion recovery
fCJD	Familial Creutzfeldt–Jakob disease
FFI	Fatal familial insomnia
GSS	Gerstmann–Sträussler–Scheinker syndrome
iCJD	Iatrogenic Creutzfeldt–Jakob disease
Keap1	Kelch-like ECH-associated protein 1
kDa	Kilodalton
MD	Molecular dynamics
Met	Methionine
MRI	Magnetic Resonance Imaging
MV	Methionine/Valine
MV1, MV2	Molecular subtypes of sCJD
MM	Methionine/Methionine
MM1, MM2	Molecular subtypes of sCJD
MM2C	MM2 cortical subtype
MM2T	MM2 thalamic subtype
NF-L	Neurofilament light chain
Nrf2	Nuclear factor erythroid 2–related factor 2
NSE	Neuron-specific enolase
PI3K	Phosphoinositide 3-kinase
PK	Proteinase K
PMCA	Protein Misfolding Cyclic Amplification
PRNP	Prion protein gene
PrP	Prion protein
PrP^C^	Cellular prion protein
PrP^res^	Protease-resistant prion protein
PrP^Sc^	Scrapie-associated prion protein
PSWC(s)	Periodic sharp wave complexes
rPrP	Recombinant prion protein
RT-QuIC	Real-time quaking-induced conversion
SARS-CoV-2	Severe acute respiratory syndrome coronavirus 2
sCJD	Sporadic Creutzfeldt–Jakob disease
sFI	Sporadic Fatal Insomnia
TSEs	Transmissible spongiform encephalopathies
Val	Valine
VV	Valine/Valine
VV1, VV2	Molecular subtypes of sCJD
VPSPr	Variably protease-sensitive prionopathy
vCJD	Variant Creutzfeldt–Jakob disease
